# Dr. James Chapman

**Published:** 1901-04

**Authors:** 


Dr. James Chapman, of Medina, died in that village February 27, 1901, aged 77 years. Dr. Chapman was a native of Orleans county, graduated at New York University in 1852, served throughout the civil war as a medical officer and then located at Medina, where he has practised his profession until his late illness.

